# Influenza A/H1N1 septic shock in a patient with systemic lupus erythematosus. A case report

**DOI:** 10.1186/1471-2334-11-358

**Published:** 2011-12-29

**Authors:** Konstantinos Tselios, Ritsa Tsioka, Alexandros Sarantopoulos, Eleni Mouloudi, Panagiota Boura

**Affiliations:** 1 Clinical Immunology Unit, 2nd Department of Internal Medicine, Hippokration General Hospital, Aristotle University of Thessaloniki, Thessaloniki, Greece; 2 Intensive Care Unit, Hippokration General Hospital, Thessaloniki, Greece

**Keywords:** Influenza A/H1N1, systemic lupus erythematosus, septic shock

## Abstract

**Background:**

Immunocompromised patients, such as systemic lupus erythematosus (SLE) sufferers have an increased risk of mortality, following influenza infection. In the recent pandemic, influenza A H1NI virus caused 18449 deaths, mainly because of adult respiratory distress syndrome or bacterial co-infections.

**Case Presentation:**

In this case report, an SLE patient with viral-induced septic shock, without overt pulmonary involvement, is discussed. The patient was administered oseltamivir and supportive treatment, including wide-spectrum antibiotics, vasopressors and steroids, according to the guidelines proposed for bacterial sepsis and septic shock. She finally survived and experienced a lupus flare soon after intensive care unit (ICU) discharge.

**Conclusions:**

To our knowledge, this is the first case to report severe septic shock from influenza A/H1N1 virus, without overt pulmonary involvement.

## Background

Infections are among the most important causes of morbidity and mortality in systemic lupus erythematosus (SLE). However, viruses are not considered to cause serious infections in these patients; they, usually, represent reactivation of herpes viruses, such as herpes simplex virus and varicella-zoster virus [1]. Nevertheless, it is reported that immunocompromised patients have an increased risk of mortality, following influenza infection [2].

In the recent pandemic, influenza A H1N1 virus has been estimated to cause approximately 18.449 deaths in 214 different countries until August 1^st ^2010 [3]. Adult respiratory distress syndrome (ARDS), along with bacterial co-infections were the direct causes of death in most cases [4]. However, no cases of viral-induced septic shock without severe pulmonary involvement have been reported. Nevertheless, little is known about this infection in SLE patients [5]. Herein, we report a case of an SLE patient, who developed septic shock due to influenza A H1N1 infection, without acute lung injury.

## Case Presentation

A 46-year old female was admitted to the hospital because of low-grade fever, sore throat and fatigue for four days; she was on clarithromycin 500 mg twice daily, as she was considered to suffer from upper respiratory tract infection by her general practitioner.

The patient had a history of SLE for 24 years, antiphospholipid syndrome and autoimmune hypothyroidism. SLE was diagnosed in the background of immune thrombocytopenic purpura (ITP), starting at the age of 8. At the age of 12, splenectomy was performed to control refractory thrombocytopenia. Currently, SLE was adequately controlled (Systemic Lupus Erythematosus Disease Activity Index, SLEDAI = 0, anti-dsDNA antibodies negative, C3 and C4 levels normal); medication included methylprednisolone 8 mg/day, azathioprine 50 mg/day, aspirin 50 mg/day and levothyroxine 150 μg/day. The patient had been vaccinated against seasonal influenza and Streptococcus pneumoniae a month before, but not against A/H1N1 virus.

**Figure 1:**
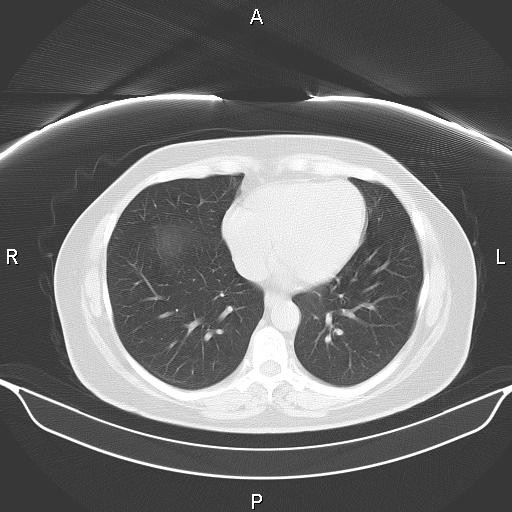
**Computed tomography of the thorax**. Thorax CT was normal on admission to the ICU, indicating the absence of overt pulmonary involvement.

On admission, she was in severe cardiovascular instability with hypotension (BP = 60/40 mmHg), tachycardia (HR = 130/min), tachypnea (RR = 30/min), hypothermia (< 35.5°C), along with oliguria and altered mental status. Oxygenation fraction PaO_2_/FiO_2 _was over 350. No obvious site of infection could be identified. The initial chest X-ray was normal, as well as computed tomography of the thorax (Figure 1). Heart ultrasound revealed mild diastolic dysfunction with preserved ejection fraction and no valvular disease. Concerning other organ involvement, there was mild prerenal azotemia (urea 94 mg/dl, creatinine 1.5 mg/dl) and severe liver impairment (alanine aminotransferase 2837 U/L, aspartate aminotransferase 3165 U/L).

The patient was considered to suffer from systemic inflammatory response syndrome (SIRS) and was treated with vigorous fluid resuscitation (crystalloids and colloids), oseltamivir 150 mg/day and moxifloxacin (400 mg/day, after appropriate cultures were obtained). A few hours later, she was intubated and carried to the ICU, because of refractory shock, where vasopressor therapy (noradrenaline up to 2 μg/kg/min) was administered to maintain mean arterial pressure ≥65 mmHg.

Arterial pressure wave form analysis (FloTrac/Vigileo System), continuous ScVO_2 _and CVP monitoring were used to estimate patient's hemodynamics. After initial fluid resuscitation, cardiac index, ScVO_2 _and CVP measurements were 2.7 L/min/m^2^, 75% and 13 mmHg, respectively (mean values). Blood lactate levels were 4.8 mmol/L. The ventilatory support consisted of controlled MV with tidal volume 7 ml/kg and respiratory rate 15/min. Initial plateau pressure was 16 cmH_2_O with respiratory system compliance 58 ml/cmH_2_O.

The patient responded after 24 hours; noradrenaline was tapered to 0.25 μg/kg/min and hydrocortisone (300 mg/day) was added as adjunctive therapy. Additional antibiotics included piperacillin/tazobactam (18 g/day) and linezolide (1.2 g/day). Blood, urine and bronco-alveolar lavage (BAL) cultures were negative. Real-time PCR for influenza A/H1N1 virus (BAL specimen) was positive and oseltamivir was administered at 300 mg/day.

In ICU, the clinical course was complicated by bacterial co-infections 10 days after admission; septicaemia due to carbapenem-resistant Klebsiella pneumoniae and ventilator-associated pneumonia due to multi-drug resistant Acinetobacter baumanii. Gentamicin and colistin were administered, according to strain sensitivity, leading to complete resolution of the lesions. She was extubated after 19 days and transferred to the ward.

Concerning H1N1 virus, BAL rt-PCR was persistently positive for 21 days. Oseltamivir was administered in high doses (300 mg/day) for 28 days and was discontinued after negative PCR.

The patient experienced a lupus flare (SLEDAI = 4, C3 = 65 mg/dl, C4 = 7.4 mg/dl), soon after extubation with thrombocytopenia and severe haemolytic anemia. The flare was successfully managed with steroids and intravenous immunoglobulins (IVIGs). Her clinical condition was complicated by severe critical illness polyneuropathy/myopathy (CIP/CIM), managed with long term physiotherapy. In four months, previous therapeutic regimen was re-established and satisfactory performance status was maintained.

## Conclusions

Influenza A/H1N1 pandemic represents a global health issue, as it was estimated to be the direct cause of over 600 million respiratory infections and over 50 million hospitalizations, until August 10^th^, when WHO announced that H1N1 was in post-pandemic period [6]. Immunosupression is a well-defined risk factor for worse outcome in H1N1 disease. ARDS and secondary bacterial super-infections, leading to septic shock and multiple organ failure are considered to be the primary causes of death [4,7]. However, primary viral septic shock, especially by influenza, is rarely reported in the literature [8-11].

The mortality pattern in SLE is reported to be biphasic; major infections play an important role during the first years of disease, while cardiovascular and other disease complications account for most deaths in long-lasting disease [12]. Viral infections, however, are not reported to affect mortality in SLE [1,13].

Severe cardiovascular instability, as indicated by the hemodynamic monitoring of the patient, with refractory shock in H1N1 infection, has not been reported so far in the literature. Acute lung injury and/or ARDS due to H1N1 virus could not be identified, as initial chest X-ray and thorax CT were normal and oxygenation was not impaired (PaO_2_/FiO_2_≥350) throughout the disease course.

Bacterial infections, as potential causes of septic shock, were not diagnosed; serial blood, urine and BAL cultures were negative. The therapeutic approach was that of conventional septic shock, according to 2008 guidelines and resulted in patient recovery [14].

Long lasting viral persistence, despite recommended oseltamivir therapy, probably reflects a poor immune status and defective natural and acquired antibacterial immunity, due to several reasons. Patients with SLE are considered to be immunocompromised either because of the disease itself or due to the immunomodulating agents used for disease management [15]. On the other hand, splenectomy is not considered to represent a major risk factor for viral infections.

Vaccination is suggested to be less effective in SLE, especially in the background of azathioprine treatment, although measurement of antibody titers can only indirectly assess the efficacy of vaccination [2]. Latest studies supported that seroprotection after a single vaccination in SLE patients was significantly reduced compared to healthy controls [16]. The presented patient was vaccinated against seasonal influenza but not against H1N1 virus. Although recent reports suggest that CD8+ T cells are able to cross react with H1N1 virus, they are functionally impaired [17].

The major complication in this patient, after ICU discharge, was a disease flare (thrombocytopenia and hemolytic anemia) along with CIP/CIM. The latter complicates approximately 30-50% of ICU patients, particularly in cases of multiple organ failure and septic shock [18]. The pathophysiologic process behind CIP/CIM is not fully elucidated; reactive oxygen intermediates, drug toxicity, steroid therapy and poorly controlled hyperglycemia are considered to be critical predisposing factors [18]. Treatment options are not well validated, although intravenous immunoglobulins seem to have a beneficial effect [19].

IVIGs were administered to this patient for managing severe autoimmune haemolytic anemia and thrombocytopenia; their benefit in recovery from CIP/CIM can not be assessed directly. However, IVIGs allowed quick steroid tapering and, possibly, prevention of further nosocomial infections.

To our knowledge, severe septic shock from influenza A/H1N1 virus, without overt pulmonary involvement, has not been reported in the literature. The authors of this case report of special interest from many points of view, feel it would be a positive step to share their experience with experts in the field. Physicians' awareness and prompt and aggressive supportive treatment are expected to optimize patient outcomes.

## Consent

Written informed consent was obtained from the patient for publication of this case report and any accompanying images. A copy of the written consent (in Greek) is available for review by the Editor-in-Chief of this Journal.

## Competing interests

The authors declare that they have no competing interests.

## Authors' contributions

KT, AS and PB were the attending physicians throughout the disease course. PB is the Head of the Clinical Immunology Unit and revised the manuscript. KT drafted the manuscript. AT and EM were the attending physicians during ICU hospitalization. All authors read and approved the final manuscript.

## Pre-publication history

The pre-publication history for this paper can be accessed here:

http://www.biomedcentral.com/1471-2334/11/358/prepub
